# The Effect of Dantonic Pill on **β**-Catenin Expression in a Rat Model of Streptozotocin-Induced Early Stage of Diabetic Nephropathy

**DOI:** 10.1155/2013/848679

**Published:** 2013-04-14

**Authors:** Zhou Shuhong, Lv Hongjun, Cui Bo, Xu Li, Shi Bingyin

**Affiliations:** Department of Endocrinology, the First Affiliated Hospital of Xi'an Jiaotong University School of Medicine, Xi'an 710061, China

## Abstract

Diabetic nephropathy (DN) is one of the most common causes of end-stage renal failure. This study was performed to determine the effect of Dantonic Pill (DP) treatment on **β**-catenin expression in a rat model of streptozotocin- (STZ-) induced early-stage DN, with irbesartan treatment as a positive control. Including an analysis of the general metabolic index and renal function, immunohistochemical staining and reverse transcription real-time PCR for **β**-catenin were performed in the renal cortex of the rat models every 4 weeks. After the treatments of DP and irbesartan, the albuminuria level, kidney weight/body weight, and thickness of the glomerular basement membrane were decreased, but the expression of **β**-catenin was not downregulated in the renal cortex. The effective drug target of DP to ameliorate albuminuria and renal hypertrophy should not inhibit the upregulated expression of **β**-catenin in rats with STZ-induced early-stage diabetic damage.

## 1. Introduction

Diabetes mellitus presents a significant health concern because this disorder leads to long-term complications throughout the body involving the renal and other systems [1]. Diabetic nephropathy (DN) evolves into a progressive fibrosing kidney disease. Wnt pathway components have been reported to be associated with various kidney diseases including DN [2]. Regulating the *β*-catenin protein levels to control the activation of Wnt-responsive target genes is referred to as the canonical Wnt/*β*-catenin pathway. Wnt proteins interact with receptor proteins and stabilize the downstream transcription regulator *β*-catenin by inhibiting *β*-catenin phosphorylation, which reportedly affects tubule formation and epithelial differentiation [3]. High glucose levels (HG) increased the phosphorylation of *β*-catenin and reduced the nuclear *β*-catenin levels. The destabilization of *β*-catenin was correlated with the increased expression of other profibrotic factors in mesangial cells [4]. Impaired *β*-catenin signaling is one prominent pathologic reaction responsible for the ECM metabolism induced by HG in mesangial cells [5].

“Herbal medicines” and ‘‘herbal remedies” are interchangeable terms that are used to refer to treatments containing various mixtures of herbs. People choose to take herbal medicines as alternatives to orthodox medicines due to their supposed low levels of toxicity and their ‘‘natural” origins. Thousands of years ofclinic practice in traditional Chinese medicine (TCM) have accumulated a considerable number of formulae that exhibit reliable in vivo efficacy and safety. The “Dantonic Pill” (DP), also known as the “Cardiotonic Pill,” has shown significant therapeutic benefits in patients with DN [6]. Although the DP has been widely used for many years, systematic scientific evidence and proof of efficacy are generally lacking compared with synthesized chemical medicines [7]. Chinese herbal formulae consist of multiple herbs and are therefore liable to produce a large number of metabolites that may act on multiple targets in the body. 

The molecular mechanisms that underlie the progression of DN to end-stage renal failure are not well defined, thus limiting access to potential therapeutic targets. Therefore, a likely therapeutic strategy may be the modulation of the *β*-catenin levels and/or function. Numerous clinical studies have defined proteinuria as a major marker of the decline of renal function and treatment with irbesartan, which reduces urinary albumin excretion, as a positive control. However, to date, whether DP has an effect on the *β*-catenin levels in early-stage DN remains unclear. This study was designed to establish diabetic animal models to observe the interventional effects of DP on *β*-catenin expression in early-stage diabetic kidney injury rats and to provide pathogenic and theoretical evidence of DN in these rats.

## 2. Materials and Methods 

### 2.1. Materials

This study was performed at the Endocrinology Department, Laboratory of the First Hospital Affiliated to Xi'an Jiaotong University from October 2009 to January 2011. Male SD rats (weighing 180–200 g; clean grade) were selected from the Experimental Animal Center of the Medical College of Xi'an Jiaotong University and cared for using standardized methods. The rats were maintained under temperature-controlled conditions (22°C ± 2°C) and artificial 12 h light/dark cycles with food and water ad libitum. All rats were randomly divided into four groups: the nondiabetic control rats with vehicle treatment (control group, *n* = 21), the streptozotocin- (STZ-) induced diabetic nephropathy rats with vehicle treatment (STZ + vehicle group, *n* = 21), the STZ-induced diabetic rats with irbesartan treatment (STZ + irbesartan group, *n* = 21), and the STZ-induced diabetic rats with DP treatment (STZ + DP group, *n* = 21). In each group, 7 rats were evaluated every 4 weeks until the end of the study, which involved a 12-week observation period. The study was approved by the Institutional Animal Ethical Committee.

### 2.2. Methods

#### 2.2.1. Model Establishment and Drug Administration

Streptozotocin (10 g/L) was single-peritoneally injected at a dose of 60 mg/kg body weight to establish the diabetic model. The blood glucose levels derived from the caudal vein were, respectively, measured at 72 h and on the 6th day after the injection. The model standard was confirmed by both of the blood glucose levels >16.6 mmo/L. The rats in the STZ + irbesartan and STZ + DP groups were administered with irbesartan 50 mg/(kg·day) and DP 500 mg/(kg·day), respectively, starting 4 weeks after model establishment. Every 4 weeks, 24-hour urine was reserved, blood was collected from the heart, and rats were sacrificed under anesthesia. Renal cortex tissue (100 mg) was used to extract total RNA, and the remaining tissues were fixed with 4% paraformaldehyde for pathological examination.

#### 2.2.2. Renal Biochemical and Functional Detection

The serum creatinine (serum Cr) concentration was determined using a commercial assay kit (BeckmanCoulter, Miami, FL, USA). The urine creatinine concentration was measured using an assay based on Jaffe's reaction according to the manufacturer's protocol (Creatinine Assay Kit). The creatinine clearance rate (Ccr) was calculated using the following equation: Ccr[ml/(min*·*kg body weight)] = [urinary Cr (*μ*mol/L) × urinary volume (ml)/serum Cr (*μ*mol/L)]/[1/1440 (min)] × [1000/body weight (g)]) [8]. The urine protein level was detected by radioimmunoassay following the manufacturer's protocol. The kidney weight/body weight (KW/BW, %) was measured.

#### 2.2.3. Renal Pathological Examination

The renal glomerulus and renal tubule mesenchymal lesions were observed following HE and PAS staining. Twenty renal glomeruli were randomly selected from the cortical area under 400x magnification by an experienced pathologist to measure the index of mesangial expansion. The index of mesangial expansion was scored by a quantitative estimate of the mesangial zone width in each glomerulus, expressed as a function of the total glomerular area [9]: 0, normal glomeruli; 1, matrix expansion occurring in up to 50% of the glomerulus; 2, matrix expansion occurring in 50–75% of the glomerulus; and 3, matrix expansion occurring in 75–100% of the glomerulus. 

#### 2.2.4. Renal Electron Microscopy Morphometric Evaluation

The electron microscopy morphometric evaluation was performed as described below. A portion of the renal tissues was cut into 1 mm cubes, fixed in 2.5% glutaraldehyde, and postfixed in 1% osmium tetroxide. The samples were dehydrated in a graded alcohol series and embedded in Epon 812. Four ultrathin sections (60 nm) were cut with a diamond knife continuously and stained with uranyl acetate and lead citrate. The thickness of glomerular basement membrane (GBM) was examined with a Hitachi H-7650 (80 kV) transmission electron microscope (JEM100SX, Japan).

#### 2.2.5. Determination of *β*-Catenin Protein Expression by Immunohistochemistry (IHC)

Paraffin sections were routinely dewaxed, hydrated, and dipped in a 0.03 volume fraction of H_2_O_2_ and methyl alcohol. Then, a microwave was used to repair the antigen, and the tissues were blocked with normal caprine serum and dribbled with attenuated antibody I (antirabbit). The rat *β*-catenin antibody was diluted at the ratio of 1 : 50, stained with biotin-labeled caprine antirabbit antibody I and diaminobenzidine, restained with hematine, dehydrated, cleared, and sealed with glycerin gelatin. Stained renal glomeruli and tubules that were randomly selected from 20 sights were observed under 400x magnification by an experienced pathologist. The positive staining rate was evaluated as the following: ≤25% positive cells (+), 26–50% positive cells (++), 51–75% positive cells (+++), and >75% positive cells (++++); each scored as 1, 2, 3, and 4 points, respectively, for the statistical analysis [10].

#### 2.2.6. Determination of *β*-Catenin mRNA Expression by Quantitative Real-Time PCR

Total RNA was extracted from the renal tissues with the Trizol method according to the manufacturer's protocol. cDNA was synthesized by reverse transcription using a kit (TaKaRa Company, Dalian, CA). The expression of *β*-catenin mRNA was detected by quantitative real-time polymerase chain reaction (RT-PCR). *β*-actin was used as the internal reference. The total volume of the PCR was 20 *μ*L, with 30 ng of cDNA as the template. After an initial denaturation step at 95°C for 3 min, 40 cycles of 95°C for 30 sec and 60°C for 30 sec for annealing and extension were run on a CFX384 Dice real-time PCR system (Bio-Rad Laboratories, Inc., CA). A melting curve analysis was performed after the amplification was completed. The mRNA levels were normalized to the *β*-actin levels of the respective control and presented as a ratio. The primers (designed and synthesized by Dalian Baosen Company) and their sequences, product lengths, and reactive conditions are shown in Table 1.

#### 2.2.7. Statistical Analysis

The measurement data were expressed as the mean ± SD. Significant differences among the groups were analyzed using a factorial-designed ANOVA. The method of simple linear correlation analysis was employed to measure the correlation between two variables. *P* values < 0.05 were considered significant. SPSS 16.0 software was used in this study.

## 3. Results

### 3.1. General Group Comparisons

There were four groups of rats in the study, and the metabolic indices, such as body weight, food intake, water intake, urine output, and blood glucose levels, for each group are summarized in Table 2. The food intake, water intake, urine output, and blood glucose levels in the rats with STZ-induced diabetes were higher thanthosein the normal control rats (*P* < 0.05). The body weights were lower in the diabetic rats than in the normal rats. However, there were no significant differences in any of the metabolic indices among the diabetic rats treated with vehicle, DP, and irbesartan during the 12 weeks (*P* > 0.05).

### 3.2. Effect of DP and Irbesartan on Renal Functional and Pathological Examination

**Figure 1:**
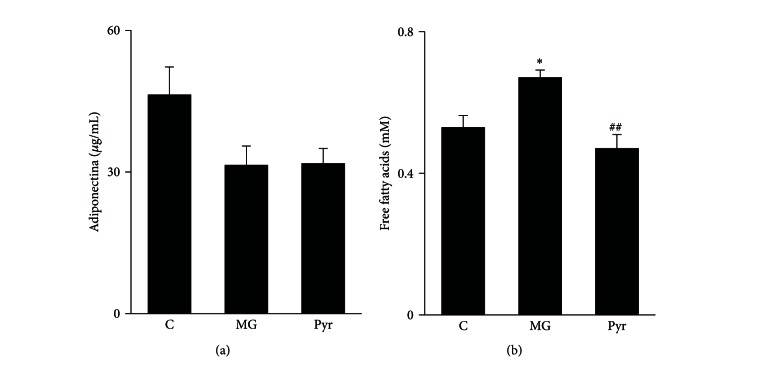
Effect of DP and irbesartan treatments on the renal pathological changes. (a) Renal pathological changes of mesangial expansion were examined by histological staining (HE and PAS), and thickness of the GBM was determined by electron microscopy, as described in the materials and methods section. (A)(E)(I) Normal renal cortex in the control group. (B)(F)(J) STZ + vehicle group. (C)(G)(K) STZ + DP (500 mg/(kg·day) group. (D)(H)(L) STZ + irbesartan (50 mg/(kg·day)) group. (b) Thickness of the GBM (A) and mesangial expansion index (B) expressed as a quantitative estimate score. All values are the means ± SD (*n* = 7). *P* < 0.05, compared with the STZ + vehicle group.

The renal pathological examination by HE and PAS staining indicated that the mesangial matrix was increased and the renal tubule was vacuolarly degenerated in the diabetic rats. The urine protein levels, Ccr, KW/BW, mesangial expansion index, and GBM thickness were significantly higher in the diabetic rats compared with the normal rats (*P* < 0.05). After 12 weeks of treatment with DP and irbesartan, compared with vehicle alone, the urine protein levels, KW/BW, and GBM thickness were decreased in the diabetic rats, but Ccr and the mesangial expansion index were not (*P* < 0.05 and *P* > 0.05, resp.). The effects of DP and irbesartan on the renal functional and pathological examination in the diabetic rats are shown in Table 3 and Figure 1.

### 3.3. Effect of DP and Irbesartan on the mRNA and Protein Expressions of *β*-Catenin

**Figure 2:**
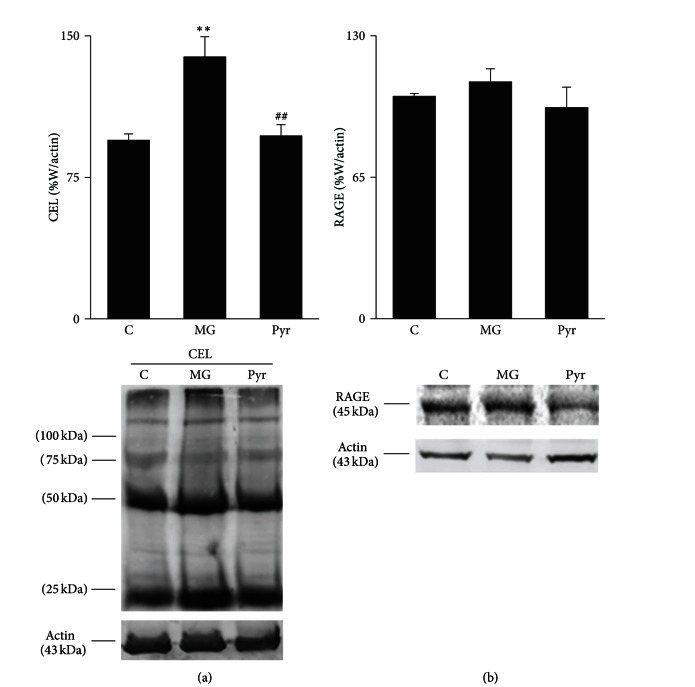
Effect of DP and irbesartan treatments on the renal expressions of *β*-catenin in the rats. (a) Effect of DP and irbesartan treatments on the renal mRNA and protein expressions of *β*-catenin as described in the materials and methods section. (A) The relative levels of the *β*-catenin mRNA were assessed by real-time PCR, and the results were normalized to *β*-actin. (B) The expression of the *β*-catenin protein expressed as a quantitative estimate score. (b) The protein expression specific to *β*-catenin ((A)–(D)) detected by immunohistochemistry (400x magnification): (A) normal glomerulus in the control; (B) STZ + vehicle group; (C) STZ + irbesartan (50 mg/(kg·day)) group; and (D) STZ + DP (500 mg/(kg·day)) group. All values are the means ± SD (*n* = 7). *P < 0.05, compared with the STZ + vehicle group.

For 12 weeks, the *β*-catenin mRNA expression in the STZ-induced diabetic rats was significantly upregulated compared with the control group (*P* < 0.05). The *β*-catenin mRNA expression in the STZ + DP and STZ + irbesartan groups was reduced, but these changes were not significantly different compared with the levels in the STZ + vehicle group (*P* > 0.05). Immunohistochemical staining of *β*-catenin in the STZ + vehicle group was greater than that of the control group (*P* < 0.05), but the staining in the STZ + DP and STZ + irbesartan groups was not less compared with the STZ + vehicle group (*P* > 0.05). The effect of DP and irbesartan on the *β*-catenin mRNA and protein expression levels is shown in Figure 2.

*Correlation Analysis*. There was no significant correlation between the mRNA and protein expression levels of *β*-catenin and the urinary protein level or the KW/BW in the diabetic rats (*P* > 0.05).

## 4. Discussion

DN is characterized by definite renal morphological and functional alterations. The features of early diabetic renal changes are glomerular hyperfiltration and glomerular and renal hypertrophy. This disease is characterized by thickening of GBM and mesangial matrix expansion [11]. The data presented here demonstrate that the long-term model of STZ-induced diabetes is associated with the early stage of diabetic nephropathy. By 16 weeks, renal hypertrophy, hyperfiltration, and proteinuria were established in our model. At that time, the earliest changes of glomerular mesangial matrix accumulation, but not collapse of the glomerular capillaries, were detectable, as previously reported [12]. In our experiments, the DP herbal preparation and irbesartan attenuated the early-stage nephropathic symptoms in the diabetic rats, which were characterized by proteinuria, kidney hypertrophy, and thickening of the glomerular basement membrane. There were no effects of the DP and irbesartan treatments over the 12 weeks on distinctive metabolic indices, especially hyperglycemia, induced in the diabetic rats. Therefore, it should be noted that these treatments possess a protective effect against the diabetic renal damage that is independent of lowering the plasma glucose level, which is in agreement with other studies [13]. In this regard, we can conclude that the therapeutic dose of DP (500 mg/(kg·day) is at least equally effective as that of irbesartan (50 mg/(kg·day) in early-stage diabetic nephropathy rats. 

The principle of formulation in TCM has been adopted to guide the selection of herbs (herb matching) in the multicomponent herbal formulae prescribed for the treatment of diseases. The most important and effective pharmacological ingredients identified in DP are danshensu and Panax notoginseng [14]. Dan Shen, as the main herb in DP, has been identified as containing lipophilic tanshinones such as tanshinone IIA and hydrophilic phenolic acids particularly including danshensu [15]. Danshensu downregulated the expression of fibronectin and collagen-1 induced by high glucose levels in human peritoneal mesothelial cells [16]. In the water extract of San Qi, Panax notoginseng downregulates the expression of *β*-tumor growth factor [17], vascular endothelial growth factor [18], and connective tissue growth factor (CTGF) [19] and inhibits the overproduction of advanced glycation end products [20] to protect the kidney in a diabetic model. Thus, we confirmed that DP therapy to treat diabetic renal complications should be effective. 

Previous studies revealed that the Wnt pathway was identified to be associated with DN. The binding of specific Wnt proteins to receptor/coreceptor complexes transduces intracellular signals through either *β*-catenin-dependent or *β*-catenin-independent pathways. There are generally two pools of *β*-catenin in cells: one that is tightly associated with cadherins at cell-cell junctions, and the other that is “free” in the cytosol/nucleus. The latter pool is involved in gene transcription regulation. In the resting state, cytosolic/nuclear *β*-catenin must be maintained at a very low level through the rapid turnover of free *β*-catenin [21]. Including the Wnt proteins, *β*-catenin activity is also regulated by tumor necrosis factor [22], N-cadherin, and matrix metalloproteinase (MMP) [23]. Moreover, ROS liberate the cadherin-sequestered pool of *β*-catenin to promote signaling [24]. The target genes of *β*-catenin are known to mediate inflammation, angiogenesis, and fibrosis through the upregulation of intercellular adhesion molecule [25], plasminogen activator inhibitor-1 [26], and CTGF [27], which are important factors participating in the pathogenesis and progression of DN [28]. Therefore, the development of STZ-induced diabetic renal disease appears to be a complex process involving *β*-catenin. 

Immunohistochemistry and real-time PCR analyses showed that the expression of *β*-catenin in the renal cortex was upregulated in our untreated diabetic rats compared with the nondiabetic control rats during the 16 weeks of this study. A similar study also showed upregulated *β*-catenin levels in both the renal cytosol and nuclei of the streptozotocin-induced diabetic rats [29]. After 12 weeks of treatment with either DP or irbesartan, the decrease in the *β*-catenin expression levels was too slight to be significantly different compared with the diabetic control rats. These results suggested that DP and irbesartan decrease urinary albumin and renal hypertrophy to prevent renal injury in the early stage of DN through a mechanism that does not involve the inhibition of *β*-catenin. The explanation is that in experimental models the kinetics concerning the development of fibrosis is faster (weeks or a few months at best) compared with that of humans (years). Therefore, the treatment in animals might have to be started relatively early, before the irreversible destruction of the renal structure (i.e., before reaching a point of no return). The molecular mechanisms that underlie the progression of DN to end-stage renal failure are not well defined, thereby limiting access to potential therapeutic targets. Because albuminuria caused by diabetic nephropathy is an independent risk factor for cardiovascular events and death [30], a strategy to protect the kidney by reducing the effects of early diabetic kidney disease is the first step in treating this disease. To the best of our knowledge, there has been no study to investigate the therapeutic mechanism of DP on DN via the regulation of *β*-catenin.

In summary, DP may contribute to the further understanding of the mechanisms of DN and to the development of novel strategies for both its prevention and management. However, our understanding of the specificities of *β*-catenin is very limited in DN due to the lack of reliable approaches to selectively assess its function. Such studies are important not only for the quality control of herbal medicine but also for the development of new pharmaceutical products in the prevention and management of diabetic nephropathy. 

## Figures and Tables

**Table 1:** Nucleotide sequence of the primers used in real-time PCR.

Gene	Primers	Nucleotide sequence 5′-3′	Length (bp)	Temperature (°C)
*β*-catenin	Forward	AACGGCTTTCGGTTGAGCTG	118	60
	Reverse	TGGCGATATCCAAGGGCTTC		
*β*-actin	Forward	TGCCTTTGTGCACTGGTATG	152	60
	Reverse	CTGGAGCAGTTTGACGACAC		

**Table 2:** Metabolic effects of STZ-induced diabetes and treatments in rats.

Parameters	Body weight (g)	Food intake (g)	Water intake (mL)	Urine output (mL)	Blood glucose (mmol/L)
0 w					
Control (*n* = 7)	443.33 ± 39.28*	24.92 ± 8.59*	40.0 ± 28.5**	10.80 ± 5.45**	4.48 ± 0.15**
STZ (*n* = 7)	303.29 ± 46.61	56.21 ± 5.25	300.03 ± 56.91	197.43 ± 42.55	28.07 ± 8.91
4 w					
Control (*n* = 7)	455.20 ± 29.54*	25.30 ± 4.6*	40.00 ± 12.51**	10.80 ± 5.45**	4.28 ± 0.35**
STZ + vehicle (n=7)	303.29 ± 46.61	56.21 ± 5.25	300.00 ± 56.92	217.43 ± 42.55	28.07 ± 8.91
STZ + DP (*n* = 7)	309.37 ± 44.57	50.28 ± 8.44	227.86 ± 38.06	171.43 ± 23.87	28.28 ± 7.18
STZ + irbesartan (*n* = 7)	346.83 ± 39.28	52.25 ± 9.80	253.43 ± 44.40	184.14 ± 34.51	24.97 ± 1.03
8 w					
Control (*n* = 7)	521.71 ± 42.59*	23.14 ± 5.81*	30.00 ± 11.00**	14.93 ± 2.46**	4.13 ± 0.41**
STZ + vehicle (*n* = 7)	331.88 ± 71.39	50.81 ± 8.27	227.50 ± 41.75	156.50 ± 20.74	28.88 ± 8.32
STZ + DP (*n* = 7)	296.29 ± 25.12	59.01 ± 7.36	232.33 ± 33.69	171.00 ± 25.82	28.33 ± 6.53
STZ + irbesartan (*n* = 7)	311.00 ± 51.78	52.25 ± 8.08	241.25 ± 54.36	178.75 ± 44.52	25.70 ± 1.28
12 w					
Control (*n* = 7)	582.33 ± 85.35*	22.33 ± 5.05*	40.00 ± 10.95**	12.35 ± 4.73**	4.16 ± 0.54**
STZ + vehicle (*n* = 7)	328.22 ± 55.96	51.77 ± 8.60	214.44 ± 44.82	160.33 ± 38.63	28.06 ± 4.87
STZ + DP (*n* = 7)	326.38 ± 54.35	53.56 ± 7.36	246.25 ± 38.89	199.38 ± 34.12	28.28 ± 1.77
STZ + irbesartan (*n* = 7)	353.00 ± 43.17	46.35 ± 4.58	232.86 ± 25.63	189.29 ± 38.90	27.20 ± 1.91

*P < 0.05 versus STZ + vehicle rats **P < 0.01 versus STZ + vehicle rats.

**Table 3:** Renal functional detection and pathological changes of STZ-induced diabetes and treatments in rats.

Parameters	Urinary protein (mg/24 h)	Ccr (mL/min·kg)	KW/BW (%)	Mesangial expansion index
0 w				
Control (*n = *7)	0.12 ± 0.07*	2.90 ± 0.47*	0.65 ± 0.09*	0.22 ± 0.02*
STZ (*n = *7)	0.32 ± 0.08	26.43 ± 7.50	1.30 ± 0.03	0.60 ± 0.02
4 w				
Control (*n = *7)	0.10 ± 0.04*	3.64 ± 1.59*	0.74 ± 0.09*	0.35 ± 0.08*
STZ + vehicle (*n = *7)	0.39 ± 0.02	13.98 ± 2.99	1.40 ± 0.03	0.64 ± 0.04
STZ + DP (*n = *7)	0.34 ± 0.02*	14.15 ± 3.31	1.26 ± 0.02*	0.46 ± 0.05
STZ + irbesartan (*n = *7)	0.20 ± 0.03*	13.51 ± 3.08	1.25 ± 0.04*	0.56 ± 0.02
8 w				
Control (*n = *7)	0.18 ± 0.02*	1.88 ± 0.46*	0.65 ± 0.04*	0.38 ± 0.08*
STZ + vehicle (*n = *7)	0.63 ± 0.04	19.40 ± 6.31	1.39 ± 0.08	0.69 ± 0.01
STZ + DP (*n = *7)	0.30 ± 0.01*	18.83 ± 2.31	1.22 ± 0.09*	0.64 ± 0.05
STZ + irbesartan (*n = *7)	0.23 ± 0.04*	17.08 ± 7.10	1.28 ± 0.06*	0.62 ± 0.02
12 w				
Control (*n = *7)	0.13 ± 0.02*	1.30 ± 0.48*	0.63 ± 0.06*	0.40 ± 0.03*
STZ + vehicle (*n = *7)	0.74 ± 0.04	15.81 ± 3.77	1.25 ± 0.06	0.79 ± 0.02
STZ + DP (*n = *7)	0.27 ± 0.05*	15.52 ± 1.50	1.20 ± 0.04*	0.68 ± 0.05
STZ + irbesartan (*n = *7)	0.26 ± 0.02*	14.19 ± 1.66	1.17 ± 0.09*	0.74 ± 0.05

*P < 0.05 versus STZ + vehicle rats.
